# Electroconvulsive Therapy for Catatonia in Autistic and Non-Autistic Patients: An Observational Study on Course, Efficacy, Aggression, and Self-Injury Outcomes

**DOI:** 10.1002/aur.70362

**Published:** 2026-09-08

**Authors:** Joshua Ryan Smith, Maria Bonnee, Sarah Marler, Seri Lim, D. Catherine Fuchs, Rafael Tamargo, Isaac Baldwin, Christopher Maley, Ashley VanHaverbeck, Courtney Hamilton, Timothy Adegoke, Haozheng Xu, Jinyuan Liu, Zachary J. Williams, Jo Ellen Wilson, James Luccarelli

**Affiliations:** 1 Division of Child and Adolescent Psychiatry, Department of Psychiatry and Behavioral Sciences, Vanderbilt University Medical Center at Village of Vanderbilt, Nashville, Tennessee, USA; 2 Vanderbilt Kennedy Center, Vanderbilt University, Nashville, Tennessee, USA; 3 Widener University, Institute of Graduate Clinical Psychology, Chester, Pennsylvania, USA; 4 Division of General Psychiatry, Department of Psychiatry and Behavioral Sciences, Vanderbilt University Medical Center at Village of Vanderbilt, Nashville, Tennessee, USA; 5 Department of Biostatistics, Vanderbilt University Medical Center, Nashville, Tennessee, USA; 6 Department of Psychiatry and Biobehavioral Sciences, Semel Institute for Neuroscience and Human Behavior, University of California, Los Angeles, Los Angeles, California, USA; 7 Department of Veterans Affairs Geriatric Research, Education and Clinical Center, Tennessee Valley Healthcare System, Nashville, Tennessee, USA; 8 Department of Psychiatry, Harvard Medical School, Boston, Massachusetts, USA; 9 Department of Psychiatry, Massachusetts General Hospital, Boston, Massachusetts, USA

## Abstract

Catatonia occurs disproportionately in autistic individuals and may respond to electroconvulsive therapy (ECT), yet prior reports suggest greater treatment burden in this population. We compared longitudinal ECT utilization, safety, and clinical outcomes between autistic and non-autistic patients with catatonia. This single-center observational cohort included 110 patients treated from May 2022 through April 2026, comprising 43 autistic and 67 non-autistic patients. Bush-Francis Catatonia Rating Scale (BFCRS), Clinical Global Impression-Improvement (CGI-I), Clinical Global Impression-Severity (CGI-S), and Kanner Catatonia Severity and Examination scores were recorded across acute and maintenance treatment. Because the cohorts differed in age, biologic sex, and intellectual disability, treatment burden was evaluated using covariate-adjusted, censoring-aware models with a three-level clinical-group variable comprising non-autism, autism with intellectual disability, and autism without intellectual disability. Autistic patients received more ECT sessions than non-autistic patients (median, 35 vs. 14) over longer observed treatment courses (median, 342 vs. 97 days). After adjustment, this difference reflected longer retention in maintenance ECT rather than a higher treatment rate and was concentrated among autistic patients with intellectual disability (Cox hazard ratio, 0.21). Clinical improvement was observed in both cohorts: CGI-I response occurred in 100.0% of autistic and 89.1% of non-autistic patients, BFCRS reduction of at least 50% occurred in 76.9% and 75.4%, and CGI-S improvement of at least 1 point occurred in 85.0% and 70.8%, respectively. Kanner total scores decreased significantly, and self-injury prevalence declined from 42.9% to 17.1% among autistic patients. Combativeness improved in both cohorts, and documented adverse events were uncommon. ECT was associated with substantial clinical improvement in autistic and non-autistic patients with catatonia. Autistic patients, particularly those with intellectual disability, experienced longer maintenance courses rather than more intensive treatment, underscoring the importance of sustained treatment access.

## 1 | Introduction

Catatonia is a psychomotor syndrome with rising recognition and high morbidity and mortality (Rogers et al. 2023; Smith, York, et al. 2024; Hirjak et al. 2024; Luccarelli et al. 2024; Luccarelli, Kalinich, et al. 2025; Luccarelli, Smith, et al. 2025; Wilson et al. 2025). While historically linked only to schizophrenia, in 2000 Wing and Shah conducted the seminal study demonstrating catatonia’s high prevalence in autism (Wing and Shah 2000). In recent years, emerging evidence suggests that catatonia may occur more frequently in autistic individuals and those with genetic disorders relative to non-autistic individuals (Baldwin et al. 2024; Shillington et al. 2023; Trelles et al. 2026). Detection of catatonia in autistic individuals is challenging given the high degree of symptom overlap with baseline features of autism and the need to assess for changes in baseline functioning (Trelles et al. 2026; Hauptman et al. 2023). The most common features of catatonia in autism are different from those of neurotypical individuals and include new-onset speech impairment, negativism, hyperactivity, recurrent self-injury, and aggression (Vaquerizo-Serrano et al. 2022; Hasan et al. 2025).

Self-injurious behaviors in autistic persons are heterogeneous and affect 40% of autistic individuals (Ferguson et al. 2025; Steenfeldt-Kristensen et al. 2020). Recent literature has suggested that recurrent self-injury and intractable aggression that do not respond to intensive behavioral interventions can be a symptom along the catatonia spectrum for autistic individuals, especially those with co-occurring intellectual disability (Wachtel et al. 2018; Wachtel 2019; Smith et al. 2025; Luiselli et al. 2021). Intensive behavioral interventions based on the principles of applied behavior analysis (ABA) (Jessel et al. 2024) remain the gold standard for aggression and self-injury in autism, often with substantial benefit (Minshawi et al. 2014; Du et al. 2024). However, catatonia-related self-injury is often deemed “automatically reinforced” on functional analysis and thus refractory to behavioral treatment, so pharmacologic and neuromodulatory options are also needed (Wachtel et al. 2018).

ECT is a safe, effective treatment for severe psychiatric disorders, including catatonia in autistic and non-autistic individuals (Espinoza and Kellner 2022; Luccarelli, McCoy, Seiner, and Henry 2021; Luccarelli, McCoy, Uchida, et al. 2021; Wachtel et al. 2025). Despite encouraging case reports and retrospective studies, research and clinical applications of ECT in catatonia and autism have been curtailed by restrictive state-dependent legislation, stigma, and limited knowledge (Espinoza and Kellner 2022; Ong et al. 2023; Sackeim 2017; Couse et al. 2024; Livingston et al. 2018). ECT providers have previously stated that, anecdotally, autistic individuals with catatonia often require a higher number of treatments over a longer period of time (Wachtel et al. 2025). This is consistent with prior reports that pharmacotherapeutic management of catatonia in autism may require higher benzodiazepine dosages and greater polypharmacy than management of catatonia in non-autistic patients (Smith et al. 2025). Whether these apparent differences reflect autism itself or are driven by co-occurring intellectual disability and other systematic differences between cohorts (e.g., age, sex, treatment era) has not been examined.

No systematic study has compared ECT treatment courses and outcomes between autistic and non-autistic patients with catatonia. To address this knowledge gap, we conducted an observational cohort study comparing catatonic patients with and without autism who received ECT at a single academic medical center. We examined whether the longitudinal course, treatment burden, and clinical response to ECT differ between autistic and non-autistic patients with catatonia, and whether any such differences persist after accounting for age, sex, treatment era, and intellectual disability.

## 2 | Methods

### 2.1 | Study Population

Utilizing *Strengthening the Reporting of Observational Studies in Epidemiology* (STROBE) guidelines (von Elm et al. 2008), we conducted an observational cohort study of patients with catatonia who received ECT at Vanderbilt University Medical Center (VUMC). Study data were collected and managed with REDCap electronic data capture tools hosted at VUMC (Harris et al. 2019, 2009). Patient information was collected longitudinally from May 11, 2022, to April 30, 2026. Patients were divided into two primary categories, those with an autism diagnosis and those without (non-autism). Patients were included if they were diagnosed with catatonia and completed an acute series of ECT, defined as receiving at least two ECT treatments at a frequency of every 7 days or less. Some patients then transitioned to maintenance ECT, which was defined as receiving treatment at a frequency of greater than every 7 days. These frequency thresholds reflect practical constraints of patients traveling long distances for care. The diagnosis of autism with or without intellectual disability was made, or confirmed if present historically, by the treating psychiatrist utilizing criteria from the Diagnostic and Statistical Manual of Mental Disorders, Fifth Edition (DSM-5) (American Psychiatric Association 2022). In both cohorts, catatonic symptom severity was assessed using the DSM-5 and/or the Bush–Francis Catatonia Rating Scale (BFCRS) (Bush et al. 1996). In the autism cohort, catatonia was also assessed via the Kanner Catatonia Rating Scale (Carroll et al. 2008). Other diagnoses which could present in a similar fashion such as anxiety, psychotic, and mood disorders were ruled out using an unstructured clinical psychiatric interview based on DSM-5 criteria by a board-certified psychiatrist.

To disentangle the effects of autism from those of co-occurring intellectual disability, we additionally classified patients into three mutually exclusive clinical groups for adjusted analyses: non-autism, autism without intellectual disability, and autism with intellectual disability. Intellectual disability was defined by clinician diagnosis and unified across cohorts using the autism-with-intellectual-disability classification for autistic patients and a standalone clinician-rated intellectual disability diagnosis for non-autistic patients.

### 2.2 | ECT Treatment

Informed consent for electroconvulsive therapy was obtained for all patients in accordance with Tennessee state law. The research study was reviewed by the VUMC Institutional Review Board (IRB #242126) and granted a waiver of informed consent, as data were collected as part of routine clinical care. This study is a continuation of our previous work investigating the use of ECT for adults and children with autism and intellectual disability (Smith et al. 2022; Smith, Baldwin, et al. 2024; Louie et al. 2024), and pediatric catatonia more broadly (Smith, York, et al. 2024, 2023; Smith et al. 2025; Smith, Baldwin, et al. 2023). However, the specific longitudinal clinic data presented here is novel and has not yet been reported.

The ECT treatments were performed using a Thymatron System IV instrument (Somatics LLC). The initial stimulus dose was determined using the half-age method (Petrides and Fink 1996) and subsequently increased as needed to achieve an adequate seizure. All treatments were performed under general anesthesia with a muscle relaxant. Of note, if ECT-induced seizures were judged inadequate in duration or quality (typically less than 20–30 s), a second stimulation was applied immediately following completion of the first seizure, as described previously (Louie et al. 2024). Because data were accrued across a 4-year interval (2022–2026) during which the program matured, we recognized that ECT administration technique could vary over calendar time. We therefore examined temporal trends in technique parameters (Table S6) and included the calendar year of ECT initiation as a covariate in all patient-level adjusted models (see Statistical Analyses).

### 2.3 | Measures

The BFCRS and Kanner Catatonia Rating Scale (Bush et al. 1996; Carroll et al. 2008) were recorded over the course of the study period. Catatonic signs and symptoms were assessed at baseline and verified with patients’ caregivers as representing a noticeable difference from the patient’s typical behavior. The BFCRS (Bush et al. 1996), the most cited rating scale in the field of catatonia (Weleff et al. 2023), includes a 14-item Bush–Francis Catatonia Screening Instrument and a 23-item severity scale. The Kanner Catatonia Rating Scale, which includes the Kanner Catatonia Severity (KCS) assessment and the Kanner Catatonia Examination (KCE), is a catatonia assessment instrument explicitly designed for assessment of catatonia in neurodivergent populations (Carroll et al. 2008; Erdoğan et al. 2022). The KCS/KCE captures catatonic symptoms not assessed by the BFCRS (e.g., self-injury, incontinence, magnetism, metronome test) and combines automatic obedience and ambitendency into a single command-verbal item for ease of administration.

Self-injury on the KCS is reported as mannerism or stereotypy with accompanying self-injury, indicated by a score of 4 or higher on each item (Carroll et al. 2008; Mahgoub et al. 2025). Thus, symptom severity was also reported in these two domains to best capture the presence of self-injury. Lastly, the clinical global impression—improvement (CGI-I) scale and clinical global impression—severity (CGI-S) (Busner and Targum 2007) scores were determined based on the patient’s improvement since starting ECT.

The BFCRS, CGI-S, and CGI-I were scored by members of the ECT team at the time the patient presented to ECT; these clinical raters were not blinded to the patient’s overall treatment course when assigning CGI-I and CGI-S values. Paired analyses required baseline and endpoint scores; longitudinal models used all available non-missing assessments.

### 2.4 | Data Collection

Demographics, co-occurring medical and psychiatric conditions, genetic syndromes, ECT parameters, calendar year of ECT initiation, location of ECT consultation, psychopharmacologic interventions, side effects of ECT, use of ketamine for intravenous access, and anesthetic and accompanying medications were recorded as part of routine ECT clinical care.

Given the heterogeneity of benzodiazepines used in the treatment of catatonia in this population, dosing of benzodiazepines was converted into lorazepam equivalents, with 0.5 mg of clonazepam, 5 mg of diazepam, and 10 mg of clobazam considered equivalent to 1 mg of lorazepam (ClinCalc.com 2024).

Cognitive adverse effects were identified from patient or caregiver reports of short-term memory difficulties or cognitive change. Formal neuropsychological testing was not administered systematically.

### 2.5 | Statistical Analyses

Statistical analyses were performed using Python (Version 3.12.4; Python Software Foundation) and R (Version 4.4.1; R Core Team, 2024). *α* was set at 0.05. Benjamini-Hochberg false discovery rate correction was applied across the six adjusted Session-by-group efficacy interaction tests, comprising two subgroup contrasts for each of BFCRS, CGI-S, and CGI-I; raw *p* values and FDR-adjusted *q*-values are reported.

Baseline differences between cohorts in the potential confounders identified during review, including age, biologic sex, and intellectual disability, were tested using Mann–Whitney *U* tests for age, chi-square tests for sex and intellectual disability, and a Mann–Whitney *U* test for benzodiazepine lorazepam-equivalent dose (Table 1). Because these characteristics differed substantially between groups, all patient-level analyses of treatment burden and efficacy were additionally adjusted for age, biologic sex, and calendar year of ECT initiation, and the cohort effect was explored in secondary analyses using the three-level clinical group (non-autism, autism without intellectual disability, autism with intellectual disability), with non-autism as the reference.

### 2.6 | Use of Generative Artificial Intelligence

Vanderbilt University Medical Center’s Microsoft 365 Copilot was used under author supervision during 2026. The tool was applied to the development, debugging, documentation, and quality review of statistical analysis code and to consistency checks across the Methods and Results sections, main tables, Supporting Information tables, and STROBE checklist. All statistical code was reviewed by the authors, executed in the authors’ controlled computing environment, and validated through inspection of model outputs, source-data checks, and reconciliation of reported results with the analytic datasets and tables. Statistical methods, interpretation of results, scientific conclusions, and final decisions regarding manuscript content were determined by the authors. The authors reviewed and approved all AI-assisted material and take full responsibility for the accuracy, originality, privacy compliance, and integrity of the final work.

### 2.7 | Efficacy and Longitudinal Change

To estimate longitudinal change, we fit linear mixed-effects models with patient-level random intercepts and fixed effects for Session (continuous), Cohort (autism vs. non-autism), and Cohort-by-Session interactions; population-averaged Poisson and negative-binomial GEE sensitivity models produced directionally concordant session effects. The primary statistical endpoint was the change in BFCRS, CGI-I, and CGI-S total scores from the first recorded score at ECT initiation until the last recorded treatment. Treatment response was defined as ever achieving the threshold during treatment (CGI-I ≤ 2; BFCRS ≥ 50% reduction from first score; CGI-S ≥ 1-point reduction from first score).

To determine whether cohort differences in the rate of symptom change were attributable to autism rather than to co-occurring intellectual disability or secular trends, we refit the mixed-effects models using the three-level clinical group and added age, biologic sex, and calendar year of ECT initiation as covariates. The Session-by-group interaction tested whether each autism subgroup differed from the non-autism reference in its per-session trajectory. All linear mixed-effects models were fit using restricted maximum likelihood.

Within each cohort, baseline-to-last BFCRS and CGI-S total scores were compared using Wilcoxon signed-rank tests (Table 3). For autism-cohort–specific behavioral outcomes (Table 4), paired first-to-last scores on the KCS total and item subscales, KCE total, and BFCRS combativeness were analyzed using the same approach. Pratt’s modification of the Wilcoxon signed-rank test was used for BFCRS combativeness because of the high proportion of zero-difference ties (Pratt 1959). Wilcoxon signed-rank tests were one-sided (alternative: less), reflecting the a priori hypothesis that clinical scores would decrease with ECT treatment; all other tests were two-sided.

### 2.8 | Self-Injury

Change in self-injury prevalence (defined as KCS Stereotypy ≥ 4 or Mannerisms ≥ 4) between the first and last assessments was evaluated using a two-sided exact McNemar test. The exact version was selected because only 11 patients had discordant first and last self-injury status. To characterize the self-injury trajectory across the full treatment course, we used all longitudinal assessments from autistic patients with at least two assessments containing sufficient Kanner item data to determine self-injury status. The primary longitudinal model was a population-averaged GEE for the binary self-injury outcome, specified with a binomial distribution, logit link, exchangeable within-patient working correlation, and robust standard errors, with repeated observations clustered by patient. As a sensitivity analysis, we fit a linear-probability mixed-effects model with a patient-level random intercept.

### 2.9 | Treatment Burden

Unadjusted total session count, treatment duration, and weekly treatment rate were compared between cohorts using Mann–Whitney *U* tests, with effect sizes quantified using Cliff’s delta (δ).

(Cliff 1993). Because patients initiated ECT across the study period, raw comparisons of total session counts and course duration were subject to unequal follow-up and right-censoring. Treatment burden was therefore additionally modeled using two complementary, censoring-aware approaches. First, the number of ECT sessions was modeled using negative-binomial regression with a logarithmic person-time offset, yielding the session rate per unit of follow-up time, and including the three-level clinical group, age, biologic sex, and calendar year of ECT initiation as covariates. Second, time to ECT discontinuation was modeled using all-cause and cause-specific Cox proportional-hazards regression with the same covariates. Patients still receiving ECT at study end were treated as censored. For the all-cause model, a hazard ratio below one indicates longer retention; for the cause-specific model, a hazard ratio below one indicates a lower instantaneous likelihood of improvement-based discharge.

### 2.10 | Treatment Era

ECT technique parameters were summarized by calendar year and tested for temporal trend (logistic or linear regression on year of procedure; Table S6).

### 2.11 | Handling of Missing Data

Consistent with STROBE guidance, analyses used available data without imputation. Paired first-to-last analyses included patients with both endpoint measurements, whereas longitudinal mixed-effects and GEE models used all available non-missing assessments. Outcome-specific denominators are reported in Tables 3 and 4.

## 3 | Results

### 3.1 | Demographics

One hundred and ten patients with catatonia received ECT during the study period (Table 1). Forty-three individuals were autistic, while 67 were not (non-autism). The autism cohort was younger (mean age 16.8 years [SD = 7.0], median 15; range 8–44) than the non-autism cohort (mean 35.1 years [SD = 17.9], median 30; range 6–79; Mann–Whitney *U p* < 0.001). Males predominated in the autism cohort (*n* = 30, 69.8%), whereas females were more common in the non-autism cohort (*n* = 38, 56.7%; chi-square *p* = 0.012). Intellectual disability was markedly more common in the autism cohort (*n* = 38, 88.4%) than in the non-autism cohort (*n* = 8, 11.9%; *p* < 0.001). The two cohorts did not differ in the calendar year of ECT initiation (median 2024 [range 2022–2026] for autism versus 2025 [2022–2026] for non-autism; *p* = 0.407).

### 3.2 | Specialty Populations

Two patients in the non-autism group became catatonic while pregnant. One patient was 24 weeks pregnant at the time of ECT initiation, the other was 27 weeks. Given the documented safety of ECT in pregnancy (Ward et al. 2018; Gandhi et al. 2023), as well as the severity of their symptoms and the risk to the fetus of untreated catatonia, ECT was pursued in close collaboration with the obstetrics and gynecology team. Overall, ECT was well tolerated and resulted in catatonic symptom resolution for both patients. In addition, two of the patients treated in the autism cohort are siblings. Their course has been previously described in the literature (Srinivasan et al. 2025).

### 3.3 | ECT Treatment Data

Across the study, 1685 ECT procedures were delivered in the autism cohort and 1138 in the non-autism cohort (Table 2). Procedure-level characteristics are shown in Table 2. On a per-patient basis, the autism cohort received more ECT treatments over a longer period and at a lower weekly rate than the non-autism cohort (Table 2). Unadjusted patient-level comparisons confirmed a higher total number of ECT procedures per patient in autism (median 35 vs. 14; Mann–Whitney *U* = 2394, *p* < 0.001; Cliff’s *δ*≈0.66, large), a longer treatment duration in autism (median 342 vs. 97 days; *U* = 2125.5, *p* < 0.001; Cliff’s *δ*≈0.48, large), and a lower weekly treatment rate in autism (median 0.8 vs. 1.4 per week; *U* = 1061, *p* = 0.02; Cliff’s *δ*≈−0.26, small).

Because these unadjusted comparisons may be confounded by the cohorts’ differing age, sex, and intellectual-disability composition and by unequal follow-up, treatment burden was re-examined using censoring-aware, covariate-adjusted models (Table 2). After adjustment for age, sex, and calendar year of ECT initiation, neither autism subgroup differed significantly from the non-autism reference in the rate of ECT sessions per unit of follow-up time (autism without intellectual disability, incidence rate ratio [IRR] 0.89, 95% CI: 0.34–2.36, *p* = 0.82; autism with intellectual disability, IRR 0.69, 95% CI: 0.42–1.13, *p* = 0.141); if anything, the point estimates were below one, indicating that autistic patients did not receive more intensive treatment per unit time. Calendar year of ECT initiation independently predicted session rate (IRR 1.33 per year, 95% CI: 1.07–1.65, *p* = 0.011), consistent with a secular increase in treatment intensity over the study period. In contrast, time-to-discontinuation analysis showed that autistic patients with intellectual disability remained in treatment markedly longer than patients in the non-autism group (Cox hazard ratio [HR] 0.21, 95% CI: 0.09–0.46, *p* < 0.001), with a directionally similar but non-significant estimate for autism without intellectual disability (HR 0.17, 95% CI: 0.02–1.38, *p* = 0.096). Taken together, these models indicate that the greater number of ECT sessions observed in autism reflects prolonged retention in maintenance treatment rather than a higher rate of treatment per unit time, and that this burden is concentrated among autistic patients with co-occurring intellectual disability. Transitions between phases were frequent; 33/37 (89.2%) autistic and 31/42 (73.8%) non-autistic patients who entered maintenance returned to acute-frequency treatment (Table 3).

By study completion, ECT treatment status differed substantially between cohorts (Table 1). In the autism cohort, the majority of patients remained in ongoing ECT at study end (*n* = 31, 72.1%), whereas most patients in the non-autism cohort discontinued treatment prior to completion (*n* = 50, 74.6%). In a cause-specific analysis, autistic patients with intellectual disability were less likely to reach improvement-based discharge than non-autistic patients (HR 0.11, 95% CI: 0.03–0.38, *p* < 0.001), supporting the interpretation that their longer courses were characterized by continued maintenance treatment. Discontinuation for lack of improvement was rare (*n* = 3) and could not be modeled reliably.

### 3.4 | Adverse Events

Adverse events are documented in Table 2. Short-term memory complaints were documented during 4 procedures in the autism cohort (0.2%) and 38 procedures in the non-autism cohort (3.3%). These values reflect routine patient or caregiver reports rather than systematic neuropsychological testing and should not be interpreted as estimates of the true incidence of cognitive effects. Aspiration pneumonia occurred in 3 procedures involving 2 patients (0.2%); all recovered without sequelae. No aspiration events were recorded in the non-autism cohort. Intramuscular ketamine to facilitate intravenous access was used more often in the autism cohort (*n* = 403, 23.9% of procedures; mean dose 127.7 mg [SD = 62.0], median 100 mg) than in the non-autism cohort (*n* = 87, 7.6%; mean 126.1 mg [SD = 49.1], median 100 mg).

### 3.5 | Treatment-Era Trends in ECT Technique

Because data spanned 4 years, we examined whether ECT technique changed over calendar time (Table S6). The proportion of procedures employing a second stimulation for an inadequate seizure rose monotonically across the study period, from 3.0% in 2022 to 29.2% in 2026 (logistic trend odds ratio 1.42 per year, *p* < 0.001), reflecting the program’s increasing use of a second stimulation. Mean stimulus energy, charge, and EEG seizure duration varied across calendar years; annual means and linear-trend *p* values are reported in Table S6. Because technique parameters changed over the study period, calendar year of ECT initiation was included as a covariate in all patient-level treatment-burden and efficacy models.

### 3.6 | Clinical Efficacy of ECT

Denominators vary across outcomes because analyses used available data without imputation; paired-analysis denominators are reported in Tables 3 and 4. Overall, clinical efficacy analyses indicated robust improvement in both cohorts. On the CGI-I, 100.0% (40/40) of autistic patients and 89.1% (57/64) of non-autistic patients ever achieved response (CGI-I ≤ 2). On the BFCRS, 76.9% (30/39) of autistic patients and 75.4% (43/57) of non-autistic patients ever achieved ≥ 50% symptom reduction. On the CGI-S, 85.0% (34/40) of autistic patients and 70.8% (46/65) of non-autistic patients ever achieved ≥ 1-point improvement. Paired Wilcoxon signed-rank tests confirmed significant baseline-to-last reductions in BFCRS and CGI-S scores in both cohorts (all *p* < 0.001; Table 3), with large effect sizes (*r* = 0.66–0.83).

Mixed-effects models (procedure-level; random intercepts for patient; fixed effects for Session, Cohort, and Cohort × Session) showed comparable clinician-rated improvement on the CGI-I (autism session *β* = −0.017, 95% CI: −0.020 to −0.014, *p* < 0.001; non-autism *β* = −0.021, 95% CI: −0.029 to −0.013, *p* < 0.001; Cohort × Session interaction *p* = 0.382). On the CGI-S, decline was steeper in non-autism (*β* = −0.058, 95% CI: −0.065 to −0.052, *p* < 0.001) than in autism (*β* = −0.008, 95% CI: −0.010 to −0.006, *p* < 0.001; interaction *p* < 0.001), and the same pattern held for the BFCRS (non-autism *β* = −0.238, 95% CI: −0.293 to −0.183; autism *β* = −0.094, 95% CI: −0.116 to −0.072; both *p* < 0.001; interaction *p* < 0.001).

To determine whether the slower per-session decline in the autism cohort was attributable to autism itself rather than to co-occurring intellectual disability or secular trends, we refit these models using the three-level clinical group with adjustment for age, sex, and calendar year of ECT initiation (Table 3). Relative to the non-autism reference (BFCRS session slope −0.242, *p* < 0.001), the autism-without-intellectual-disability subgroup had a nominally faster BFCRS decline (slope −0.494; raw interaction *p* = 0.043, FDR *q* = 0.086), but this finding did not survive FDR correction and was based on only five patients. BFCRS decline was attenuated in autism with intellectual disability (slope −0.093; raw interaction *p* < 0.001, FDR *q* < 0.001). A parallel pattern was observed for CGI-S: decline was attenuated in autism with intellectual disability (non-autism slope −0.059; autism with intellectual disability slope −0.008; raw interaction *p* < 0.001, FDR *q* < 0.001), whereas the autism-without-intellectual-disability interaction was not significant (slope −0.042; raw *p* = 0.107, FDR *q* = 0.161). CGI-I trajectories did not differ significantly for autism with intellectual disability (raw interaction *p* = 0.293, FDR *q* = 0.352) or autism without intellectual disability (raw interaction *p* = 0.953, FDR *q* = 0.953). These adjusted analyses indicate that the more gradual symptom trajectory in autism was concentrated among patients with co-occurring intellectual disability, whereas estimates for autism without intellectual disability were exploratory because this subgroup included only five patients. Clinician-rated global improvement on CGI-I proceeded at a comparable rate across all three groups. Combativeness improved significantly in both cohorts (Table 3).

### 3.7 | Autism-Cohort–Specific Outcomes

Among autistic patients with paired first and last assessments (*n* = 35), the KCS total score decreased significantly from 26.97 (SD = 12.71) to 16.11 (SD = 10.91), a mean reduction of −10.86 points (SD = 11.43; Wilcoxon *p* < 0.001, *r* = 0.72), with 88.6% of patients demonstrating any reduction (Table 4). On examination-based measures (*n* = 36), the KCE total score decreased from 2.11 (SD = 1.09) to 0.89 (SD = 0.85), a mean reduction of −1.22 points (SD = 1.20; *p* < 0.001, *r* = 0.76), with 75.0% of patients showing improvement. Linear mixed-effects models confirmed significant session-wise declines in both KCS total (*β* = −0.168, 95% CI: −0.211 to −0.125; *p* < 0.001) and KCE total scores (*β* = −0.017, 95% CI: −0.022 to −0.013; *p* < 0.001).

Self-injurious behavior, defined as a score ≥ 4 on either KCS Stereotypy or KCS Mannerisms, was present in 15 of 35 evaluable patients (42.9%) at first assessment and in 6 of 35 patients (17.1%) at last assessment, an absolute reduction of 25.7 percentage points (McNemar’s exact test, *p* = 0.012). Of 11 discordant cases, 10 reflected improvement (self-injury present → absent) and one reflected worsening (absent → present). This endpoint reduction was evaluated longitudinally using 451 assessments from 35 autistic patients with at least two assessments containing sufficient Kanner item data to determine self-injury status. In the longitudinal GEE model, the odds of self-injury decreased by an estimated 2.4% per session, although the association did not meet the prespecified significance threshold (OR 0.976 per session, *p* = 0.058).

The linear-probability mixed-effects sensitivity model showed a decline of 0.4 percentage points per session (*β* = −0.004, *p* < 0.001). Both estimates were directionally consistent with the significant first-to-last McNemar result. Fifteen autistic patients (34.9%) were receiving ABA at ECT onset; self-injury prevalence declined over treatment in the cohort as a whole, and the small number of patients with self-injury within ABA-defined subgroups precluded a reliable stratified inferential comparison.

## 4 | Discussion

This study compares ECT treatment response between autistic and non-autistic individuals with catatonia. ECT was well tolerated and associated with significant improvements in catatonia severity in both groups. To our knowledge, this paper represents the largest dataset on the use of maintenance ECT in catatonia for any condition (Gil-Badenes et al. 2024) and provides the first statistically rigorous group-level evidence of effective treatment for catatonia-related self-injury and aggression in autistic patients. Although prior case reports and clinical reviews have described resolution of self-injury with ECT in autistic individuals with catatonia (Wachtel et al. 2018, 2025; Luiselli et al. 2021), no prior study has demonstrated this finding using formal statistical testing. Moreover, pharmacological options for treatment-refractory self-injury similarly remain limited, with a recent Cochrane review finding only low-quality evidence for available agents (Iffland et al. 2023). Deep brain stimulation has shown preliminary promise in a Phase I pilot trial of six children (Gorodetsky et al. 2024), but remains investigational. Our data suggest that when recurrent self-injury and aggression occur in the context of catatonia, ECT offers a viable and effective treatment pathway for symptoms that have historically been considered treatment-resistant, with significant implications for autistic individuals, their families, and caregivers (Mandell 2008; Biswas et al. 2023).

Autistic patients with catatonia required more ECT sessions over a longer period of time relative to non-autistic individuals with catatonia. Relative to the non-autism group, autistic patients improved more slowly with each ECT session and more often returned to acute ECT treatment after entering the maintenance phase (89.2% vs. 73.8%). However, overall response rates were similar and numerically higher in the autism group across all three outcome measures, with no significant between-group differences in CGI-I trajectory (interaction *p* = 0.382), suggesting that the need for more sessions does not diminish the maximal efficacy of ECT for autistic patients. After adjustment, this greater burden reflected prolonged retention (all-cause HR 0.21) and a lower likelihood of improvement-based discharge (cause-specific HR 0.11), rather than greater treatment intensity per unit time; these findings were concentrated in autism with intellectual disability.

Autistic patients also more often remained in active ECT at study end, whereas non-autistic patients more often discontinued maintenance care. These findings have critical implications for how clinicians conceptualize ECT use in catatonia. Historic research has found that generally fewer than 10 ECT treatments are delivered on average for the treatment of catatonia (Rogers et al. 2023; Leroy et al. 2018) regardless of neurodivergence. Our reported numbers of total ECT treatments are much higher for both the autism and non-autism groups; they are, however, inclusive of maintenance ECT, which has not been the case in previous reports on ECT in catatonia.

Clinically, we found that patients in both cohorts improved significantly across the study period. Autism-specific analyses showed significant reductions in KCS and KCE total scores and in BFCRS combativeness (Tables 3 and 4). Most notably, clinically significant self-injury fell from 42.9% to 17.1% (McNemar *p* = 0.012; Table 4).

Adverse events were infrequently reported. However, cognitive and subjective adverse effects were difficult to assess systematically in this population. Formal neuropsychological testing was not administered systematically and documented short-term memory complaints were based on patient or caregiver report. Severe catatonia, co-occurring intellectual disability, baseline communication limitations, and active symptoms at the time of presentation for ECT frequently limited the feasibility of conventional cognitive testing. Consequently, the low frequency of documented short-term memory complaints should not be interpreted as evidence that cognitive effects were absent or as an estimate of their true incidence. Future prospective studies should incorporate developmentally appropriate and communication-accessible cognitive measures, while also assessing caregiver-reported changes in memory and day-to-day functioning while receiving ECT. We observed a relatively high rate of prolonged seizures requiring propofol (*n* = 39, 2.3% of procedures in the autism cohort; *n* = 42, 3.7% in the non-autism cohort), possibly reflecting the overrepresentation of pediatric patients in the autism cohort (Smith, Baldwin, et al. 2024; Isenberg et al. 2024; Karl et al. 2022; Puffer et al. 2016; Ghaziuddin et al. 2020). Autistic patients more often received ECT in the outpatient setting, consistent with prior findings that neurodivergent children hospitalized for catatonia have shorter admissions and less clinical improvement (Luccarelli, Clauss, et al. 2025).

Catatonia patients in both cohorts frequently required multiple stimulations of ECT for inadequate seizure length, likely due to the high degree of benzodiazepine and antiepileptic use in the treatment of catatonia. Newer case reports and smaller studies have shown efficacy of repeated stimulations in cases of refractory catatonia and status epilepticus (Louie et al. 2024; Abbott et al. 2024; Woodward et al. 2023; Chrobak et al. 2023).

The prevalence of autism with intellectual disability in this catatonia cohort is similar to those previously reported in the literature, and much higher in the autism cohort than in the non-autism cohort. Previous meta-analytic work from Vaquerizo-Serrano and colleagues (Vaquerizo-Serrano et al. 2022) suggested that the rate of intellectual disability for autistic individuals with catatonia is between 5.7% and 81.6%, but reaches nearly 90% in clinical samples such as this one. Given the high degree of correlation between social–emotional impairment and intellectual disability in autism (Bernard Paulais et al. 2019), it is difficult to fully differentiate these co-occurring symptoms to determine which may be the primary risk factor for catatonia in autism. It has also been speculated that genetic syndromes (Shillington et al. 2023; Raffin et al. 2018; Moore et al. 2022) may increase risk for catatonia (Fink and Taylor 2006; Dhossche et al. 2012), which were present in both the autism and non-autism cohort. Adjusted estimates localized the greater treatment burden and more gradual symptom decline to autism with intellectual disability. Estimates for autism without intellectual disability were exploratory because this subgroup included only five patients.

One possible neurobiological explanation for autistic patients requiring more ECT treatments over a longer period of time is altered cortical plasticity within the excitatory/inhibitory (E:I) imbalance model of autism, first proposed by Rubenstein and Merzenich (Rubenstein and Merzenich 2003). A prominent mechanistic instantiation of this model attributes the E:I shift to a reduction in parvalbumin-positive (PV+) GABAergic interneurons rather than to lower parvalbumin protein levels (Yao and Li 2024; Jannati et al. 2021; Hashemi et al. 2017; Contractor et al. 2021). In support of this hypothesis, recent diagnostic work in transcranial magnetic stimulation of intellectually capable persons with autism has reported enhanced cortical modulation, indicative of cortical hyperplasticity (Jannati et al. 2022). Furthermore, recent preliminary magnetoencephalographic research has reported greater E:I imbalance in biologically male profoundly impaired autistic individuals compared to autistic individuals with intact cognitive abilities (Manyukhina et al. 2022), indicative of a direct correlation between the degree of E:I imbalance and severe symptomatology in autism.

These findings, along with the proposed role of GABAergic signaling dysfunction and E:I imbalance in catatonia (Moyal et al. 2026), may explain the higher rates of catatonia and treatment refractoriness observed in autistic persons with intellectual disability. That the greatest E:I elevation has been reported specifically in autistic individuals with below-average cognitive ability (Manyukhina et al. 2022) parallels our finding that treatment burden and maintenance need were concentrated in the autism-with-intellectual-disability subgroup, and we hypothesize that a more severe cortical E:I imbalance in this subgroup may underlie a relapsing course requiring sustained treatment; this remains a hypothesis for prospective study incorporating direct E:I biomarkers.

Strengths include the large sample size, the large number of ECT procedures, prolonged follow-up, and the real-world observational design tracking outcomes across treatment. Limitations of this paper include the single-site study design, unblinded clinical raters, open-label study design, the use of routine clinical data, and the lack of a control arm. In addition, some patients received ABA, and all were receiving pharmacotherapy while they were receiving ECT. Thus, we cannot account for the influence that these therapies may have had in the recovery of patients in this manuscript. Subgroup analysis of self-injury outcomes stratified by ABA receipt was limited by small sample sizes and should be interpreted descriptively. Because intellectual disability was present in 88% of autistic but only 12% of non-autistic patients, autism and intellectual disability could not be fully disentangled, and the autism-without-intellectual-disability subgroup (*n* = 5) was too small to isolate an autism-specific effect. Another limitation includes the challenges in assessing subjective side effects in patients with limited communication abilities. This is particularly important given variable state regulation of pediatric ECT and professional calls to preserve access for critically ill or neurodevelopmentally-impaired individuals (Couse et al. 2024; Livingston et al. 2018; Zilles-Wegner et al. 2026; Lowe and Smith 2026; AACAP 2026).

Lastly, neither the BFCRS nor the Kanner Catatonia Rating Scale has been formally validated in autistic patients or children with catatonia. However, both have demonstrated strong psychometric properties in general psychiatric populations that include patients with neurodevelopmental disorders (Bush et al. 1996; Carroll et al. 2008; Erdoğan et al. 2022), and the Kanner scale was designed specifically to capture catatonic features relevant to neurodivergent persons experiencing catatonia (Carroll et al. 2008). Overall, we are hopeful that future studies will conduct controlled trials to fully investigate the efficacy of ECT for catatonia broadly, as well as catatonic self-injury and aggression in these populations, and further work to best address side effect monitoring in this population.

## Supplementary Material

Supplemental Table 1

Supplemental Table 3

Supplemental Table 2

Supplemental Table 4

Supplemental Table 5

Supplemental Table 6

STROBE Checklist

Supporting Information

Additional supporting information can be found online in the Supporting Information section. **Data S1:** STROBE statement—checklist of items that should be included in reports of observational studies. **Table S1**: Electroconvulsive therapy stimulation parameters. **Table S2**: Medications administered during electroconvulsive therapy. **Table S3**: Comorbidities for autistic patients with catatonia. **Table S4**: Comorbidities in non-autistic patients with catatonia. **Table S5**: Pharmacotherapy at the time of electroconvulsive therapy consultation. **Table S6**: Electroconvulsive therapy technique parameters by calendar year.

## Figures and Tables

**Table 1:** Demographics and Clinical Characteristics

	Autism and catatonia		Non-autism and catatonia		p^b^
	N	%	N	%	
N	43		67		
**Age (years)**					
Mean	16.8 (SD=7.0)		35.1 (SD=17.9)		<0.001
Median	15		30		
Range	8 – 44		6 – 79		
**Biologic sex**					
Male	30	69.8	29	43.3	0.012
Female	13	30.2	38	56.7	
**Intellectual disability** ^c^					
Present	38	88.4	8	11.9	<0.001
**Year of ECT initiation** ^d^					
Median [range]	2024 [2022–2026]		2025 [2022–2026]		0.407
**Location of ECT consultation**					
Inpatient	10	23.3	57	85.1	
Outpatient	33	76.7	10	14.9	
**Race and ethnicity** ^e^					
White	35	81.4	40	59.7	
Black	4	9.3	20	29.9	
Hispanic	1	2.3	6	9.0	
Asian	2	4.7	—	—	
Arabic	1	2.3	2	3.0	
Other	1	2.3	1	1.5	
**Ability to report side effects at ECT consultation**					
Cannot report – baseline NDD	18	41.9	4	6.0	
Cannot report – catatonic symptoms	17	39.5	34	50.7	
Can endorse side effects	8	18.6	29	43.3	
**Benzodiazepine prescriptions**					
Prescribed daily benzodiazepine	38	88.4	57	85.1	
Mean daily dose (LZP-eq)^f^	10.9 mg (SD=8.8)		6.6 mg (SD=5.1)		0.013
Median daily dose	8.0 mg		6.0 mg		
**ECT ongoing vs discontinued**					
Treatment ongoing	31	72.1	17	25.4	
Treatment discontinued	12	27.9	50	74.6	
Due to clinical improvement	3	7.0	33	49.3	
Due to lack of improvement	3	7.0	—	—	
Due to family relocation	1	2.3	1	1.5	
Patient lost to follow up	—	—	8	11.9	
Patient/caregiver preference/transportation challenges	4	9.3	6	9.0	
Transitioned to another ECT team	1	2.3	2	3.0	

a Percentages computed among patients with evaluable data (available-case analysis).

b p-values: age and year of initiation, Mann–Whitney U; sex and intellectual disability, chi-square.

c Intellectual disability was defined by clinician diagnosis, using the autism-with-intellectual disability classification for autistic patients and a standalone clinician-rated intellectual disability diagnosis in non-autistic patients.

d Calendar year of ECT initiation was included as a covariate in adjusted patient-level models; procedure-level ECT parameters by calendar year are presented in Supplemental Table 6.

e Race and ethnicity categories are not mutually exclusive.

f Lorazepam-equivalent daily doses were calculated among patients prescribed a daily benzodiazepine. Between-cohort comparison used a Mann–Whitney U test (U=1408.5, p=0.013).

**Table 2:** Electroconvulsive Therapy Procedures and Treatment Burden

	Autism and catatonia		Non-autism and catatonia	
	N	%	N	%
Total number of ECT procedures	1,685		1,138	
**Location of ECT procedure**				
Outpatient	1,539	91.3	659	57.9
Inpatient	146	8.7	479	42.1
**Electrode placement**				
Bitemporal	1,685	100	1,127	99.0
Right unilateral	0	0	11	1.0
**Total number of stimulations**				
One	1,204	71.5	1,025	90.1
Two	467	27.7	105	9.2
Three – titration	12	0.7	6	0.5
Four – titration	2	0.1	2	0.2
**ECT treatment metrics per patient**				
Mean total ECT procedures	39.2 (SD=21.5)		17.0 (SD=11.5)	
Median ECT procedures	35		14	
Mean treatment duration	413.7 d (SD=330.2)		202.7 d (SD=249.6)	
Median treatment duration	342 d		97 d	
Mean treatment rate	1.0 procedures/week		1.7 procedures/week	
Median treatment rate	0.8 procedures/week		1.4 procedures/week	
**Acute vs maintenance ECT** ^b^				
Total acute treatments (≤7 d)	643	38.2	688	60.5
Total maintenance treatments (>7 d)	1,042	61.8	450	39.5
**Adverse events recorded during ECT treatment** ^a^				
Headache	64	3.8	60	5.3
Prolonged seizure requiring propofol	39	2.3	42	3.7
Fatigue	32	1.9	36	3.2
Generalized muscular pain	14	0.8	18	1.6
Aspiration pneumonia	3	0.2	—	—
Short-term memory complaints^c^	4	0.2	38	3.3
**Use of IM ketamine for IV placement**				
Frequency	403	23.9	87	7.6
Mean dose of ketamine	127.7 mg (SD=62.0)		126.1 mg (SD=49.1)	
Median dose of ketamine	100 mg		100 mg	
**Covariate-adjusted treatment burden (ref = non-autism)** ^d^				
**Model / contrast**	**Estimate [95% CI]**		**p**	
Session rate IRR – Autism without ID	0.89 [0.34–2.36]		0.82	
Session rate IRR – Autism with ID	0.69 [0.42–1.13]		0.141	
Session rate IRR – per calendar year	1.33 [1.07–1.65]		0.011	
Retention HR – Autism without ID	0.17 [0.02–1.38]		0.096	
Retention HR – Autism with ID	0.21 [0.09–0.46]		<0.001	
Cause-specific HR (improvement) – Autism with ID	0.11 [0.03–0.38]		<0.001	

a Adverse-event percentages computed per procedure, not per patient.

b Acute treatment defined as inter-treatment interval ≤7 days; maintenance >7 days.

c Cognitive adverse events were identified from the reports of patients or caregivers and were not assessed using standardized neuropsychological testing.

d Adjusted models included the three-level clinical group, age, biologic sex, and calendar year of ECT initiation. Session rates were estimated using negative-binomial regression with a log person-time offset and are reported as incidence rate ratios (IRRs). Time to ECT discontinuation was analyzed using all-cause Cox proportional-hazards regression; HR<1 indicates longer retention in treatment. In the cause-specific model, discontinuation due to clinical improvement was the event; HR<1 indicates a lower instantaneous likelihood of improvement-based discharge. Non-autism was the reference group. The autism-without-intellectual-disability subgroup included five patients, and estimates should be interpreted cautiously.

**Table 3:** Clinical Efficacy of Electroconvulsive Therapy in Autistic and Non-Autistic Patients With Catatonia

	Autism and catatonia	Non-autism and catatonia
N	43	67
**Clinical Global Impression – Improvement (CGI-I)**		
Data available for CGI-I	40/43 (93.0%)	64/67 (95.5%)
Responders (score ≤2)	40/40 (100.0%)	57/64 (89.1%)
Median sessions to response	7	6
First CGI-I, mean (SD)	3.50 (0.88)	3.53 (0.80)
Last CGI-I, mean (SD)	2.17 (0.90)	2.31 (0.94)
Wilcoxon first vs last	W=36.5, r=0.70, p<0.001	W=75.0, r=0.74, p<0.001
Last CGI-I vs no change (4)	W=8.0, r=0.88, p<0.001	W=8.0, r=0.83, p<0.001
**Bush-Francis Catatonia Rating Scale (BFCRS)**		
Data available for BFCRS	39/43 (90.7%)	57/67 (85.1%)
Responders (≥50% reduction)	30/39 (76.9%)	43/57 (75.4%)
Median sessions to response	10	5
Mean baseline BFCRS (SD)	15.92 (6.58)	10.02 (6.73)
Mean last recorded BFCRS (SD)	7.10 (5.04)	3.65 (3.69)
Wilcoxon first vs last	W=12.5, r=0.83, p<0.001	W=67.5, r=0.74, p<0.001
**Clinical Global Impression – Severity (CGI-S)**		
Data available for CGI-S	40/43 (93.0%)	65/67 (97.0%)
Responders (≥1-point reduction)	34/40 (85.0%)	46/65 (70.8%)
Median sessions to response	11	5
Mean baseline CGI-S (SD)	6.33 (0.86)	6.31 (0.97)
Mean last recorded CGI-S (SD)	5.45 (0.85)	4.98 (1.34)
Wilcoxon first vs last	W=50.0, r=0.66, p<0.001	W=29.0, r=0.66, p<0.001
**Combativeness subdomain (0–3 scale)**		
Evaluable patients	39/43	57/67
Mean baseline (SD)	0.87 (1.03)	0.19 (0.58)
Mean last (SD)	0.31 (0.66)	0.02 (0.13)
Any reduction	18/39 (46.2%)	7/57 (12.3%)
Pratt first vs last^a^	r=0.52, p<0.001	r=0.35, p=0.004
**Treatment course**		
Returned to acute after maintenance	33/37 (89.2%)	31/42 (73.8%)
**Linear mixed-effects models: session-wise change (two-cohort)** ^b,e^		
CGI-I slope per session	−0.017 (−0.020, −0.014); p<0.001	−0.021 (−0.029, −0.013); p<0.001
CGI-I Cohort×Session interaction	p=0.382	
CGI-S slope per session	−0.008 (−0.010, −0.006); p<0.001	−0.058 (−0.065, −0.052); p<0.001
CGI-S Cohort×Session interaction	p<0.001	
BFCRS slope per session	−0.094 (−0.116, −0.072); p<0.001	−0.238 (−0.293, −0.183); p<0.001
BFCRS Cohort×Session interaction	p<0.001	
**Covariate-adjusted session slopes, three-level group (reference = non-autism)** ^c,d,e^		
**Measure (non-autism slope)**	**Autism without ID**	**Autism with ID**
BFCRS (non-autism −0.242; p<0.001)	−0.494; raw interaction p=0.043; FDR q=0.086	−0.093; raw interaction p<0.001; FDR q<0.001
CGI-S (non-autism −0.059; p<0.001)	−0.042; raw interaction p=0.107; FDR q=0.161	−0.008; raw interaction p<0.001; FDR q<0.001
CGI-I (non-autism −0.022; p<0.001)	−0.022; raw interaction p=0.953; FDR q=0.953	−0.018; raw interaction p=0.293; FDR q=0.352

Effect size r = |Z| / √N. Wilcoxon signed-rank tests are one-sided (alternative: less).

a Pratt’s modification of the Wilcoxon signed-rank test was used for the combativeness subdomain because of the high proportion of zero-difference ties (46.2% in autism and 87.7% in non-autism); W is omitted because the rank-sum statistic is difficult to interpret in the presence of extensive zero differences.

b Two-cohort models were fit using restricted maximum likelihood and included patient-level random intercepts and a Session × Cohort interaction. Interaction p-values test whether the per-session slope differs between the autism and non-autism cohorts.

c Adjusted three-level models were fit using restricted maximum likelihood and included Session × three-level clinical group, age, biologic sex, and calendar year of ECT initiation, with patient-level random intercepts. Cells show group-specific slopes; raw interaction p-values test differences from the non-autism reference. Autism without intellectual disability included five patients; estimates for this subgroup are exploratory and should be interpreted cautiously.

d Benjamini–Hochberg FDR correction was applied across the six adjusted Session-by-group efficacy interaction tests, comprising two subgroup contrasts for each of BFCRS, CGI-S, and CGI-I. Raw p-values and FDR-adjusted q-values are shown in the adjusted three-level panel.

e Mixed-effects slopes are expressed per ECT session; the equivalent change per 10 sessions is ten times the tabulated value (e.g., BFCRS non-autism −0.238 per session ≈ −2.38 points per 10 sessions).

**Table 4:** Changes in Catatonia-Related Behavioral Domains in the Autism Cohort

Domain	n^a^	First, Mean (SD)	Last, Mean (SD)	Change (SD)	p^b^	% Any Red.	r^c^
**KCS – Severity Items**							
KCS Total	35	26.97 (12.71)	16.11 (10.91)	−10.86 (11.43)	<0.001	88.6	0.72
KCS Stereotypy^d^	35	2.5 (1.7)	1.5 (1.5)	−1.0 (1.6)	<0.001	42.9	0.53
KCS Mannerisms^d^	35	2.1 (2.1)	1.5 (1.5)	−0.5 (1.7)	0.042	25.7	0.29
KCS Aggression^e^	35	1.77 (2.16)	1.09 (1.48)	−0.69 (2.11)	0.033	37.1	0.31
**KCE – Examination**							
KCE Total	36	2.11 (1.09)	0.89 (0.85)	−1.22 (1.20)	<0.001	75.0	0.76
**Self-Injury Prevalence** ^d,f^							
Present (Stereotypy ≥4 OR Mannerisms ≥4)	35	15 (42.9%)	6 (17.1%)	− 25.7 percentage points	0.012	—	—
**Linear mixed-effects models (autism only)**							
KCS Total slope/session	—	—	—	β=−0.168 (−0.211, −0.125)	<0.001	—	—
KCE Total slope/session	—	—	—	β=−0.017 (−0.022, −0.013)	<0.001	—	—
**Self-injury over treatment course** ^g^							
Population-averaged GEE (per session)	35	451 assessments	—	OR=0.976 (95% CI 0.951–1.001)	0.058	—	—
Linear-probability mixed model	35	451 assessments	—	β=−0.004 (95% CI −0.006 to −0.002)	<0.001	—	—

a n = patients with a complete-scale score at both a first and last assessment (≥2 paired complete assessments; complete-case, no imputation). KCS totals require all 18 severity items; KCE totals require all 12 examination items.

b Wilcoxon signed-rank test (one-sided, alternative: less) unless otherwise noted.

c Effect size r = |Z| / √N.

d Kanner items scored 0–8; ≥4 indicates self-injurious behavior.

e KCS Aggression corresponds to the KCS Combativeness item and is scored from 0 to 8. The paired comparison used a one-sided Wilcoxon signed-rank test consistent with the prespecified directional hypothesis.

f McNemar’s exact test was two-sided; the exact version was used because there were 11 discordant pairs, of which 10 reflected self-injury changing from present to absent and one reflected self-injury changing from absent to present.

g Longitudinal self-injury analyses used 451 assessments from 35 autistic patients with at least two assessments containing sufficient Kanner item data to determine self-injury status. The primary longitudinal model was a population-averaged GEE specified with a binomial distribution, logit link, exchangeable within-patient working correlation, patient clustering, and robust standard errors. The GEE estimated a 2.4% decrease in self-injury odds per session (OR=0.976, 95% CI 0.951–1.001, p=0.058). The linear-probability mixed-effects model used the same analytic sample and included a patient-level random intercept as a sensitivity analysis. Both longitudinal estimates were directionally consistent with the first-to-last McNemar result

## Data Availability

De-identified data for this study is available upon reasonable request.

## Summary

Electroconvulsive therapy (ECT) is an established treatment for catatonia, a condition that can cause severe difficulties with movement, speech, and behavior. We studied 110 patients with catatonia, 43 autistic and 67 non-autistic, who received ECT at a single center. Autistic patients received more ECT treatments over a longer period than non-autistic patients. However, when we accounted for differences between the groups, such as age and co-occurring intellectual disability, this reflected a need for longer ongoing (maintenance) treatment to stay well, rather than more intensive initial acute treatment. This longer maintenance course was mainly seen in autistic patients who also had intellectual disability. Importantly, ECT worked well in both groups and was associated with significant reductions in self-injurious behavior and aggression in autistic patients, symptoms that are often resistant to other treatments. ECT was safe and well tolerated. These findings support sustained, long-term access to ECT for autistic patients with catatonia.

## Abbreviations:

ABA: applied behavior analysis
BFCRS: Bush–Francis Catatonia Rating Scale
CGI–I: Clinical Global Impression–Improvement
CGI–S: Clinical Global Impression–Severity
DSM-5: Diagnostic and Statistical Manual of Mental Disorders, Fifth Edition
ECT: electroconvulsive therapy
E:I: excitatory/inhibitory
KCE: Kanner Catatonia Examination
KCS: Kanner Catatonia Severity
STROBE: Strengthening the Reporting of Observational Studies in Epidemiology
VUMC: Vanderbilt University Medical Center
δ: delta
