# Supplementary material for: Electroconvulsive Therapy for Catatonia in Autistic and Non-Autistic Patients: An Observational Study on Course, Efficacy, Aggression, and Self-Injury Outcomes
Source: Autism Res. Author manuscript; Available in PMC 2026 Sep 27. (PMC13616504; doi:10.1002/aur.70362)
Supplement: Supplementary file 2 [file NIHMS2209361-supplement-Supplemental_Table_1.docx]

**Supplemental Table 1: Electroconvulsive Therapy Stimulation Parameters**

|  | **Autism and catatonia** | **Non-autism and catatonia** |
| --- | --- | --- |
| **Hyperventilation performed^a^** | | |
| Yes | 619 (38.0%) | 174 (15.6%) |
| No | 1,011 (62.0%) | 942 (84.4%) |
| **First Stimulation** | | |
| Total procedures | 1,685 | 1,138 |
| Mean percentage energy | 75.3% (SD=41.7) | 60.8% (SD=52.4) |
| Mean charge | 373.2 mC (SD=186.6) | 302.2 mC (SD=196.7) |
| Mean current | 1.8 A (SD=19.9) | 1.7 A (SD=18.2) |
| Mean stimulus duration | 7.1 s (SD=3.4) | 7.2 s (SD=15.0) |
| Mean frequency | 54.4 Hz (SD=29.1) | 45.7 Hz (SD=29.5) |
| Mean pulse width | 0.8 ms (SD=12.0) | 0.6 ms (SD=2.6) |
| Mean static impedance | 1,027.5 Ω (SD=460.3) | 1,101.4 Ω (SD=728.5) |
| Mean dynamic impedance | 172.5 Ω (SD=55.4) | 176.8 Ω (SD=57.7) |
| Mean motor seizure duration | 20.5 s (SD=47.8) | 24.6 s (SD=24.3) |
| Mean EEG seizure duration | 44.2 s (SD=42.3) | 54.7 s (SD=43.6) |
| **Second Stimulation** | | |
| Total procedures | 481 | 113 |
| Mean percentage energy | 92.8% (SD=47.9) | 72.4% (SD=31.8) |
| Mean charge | 465.9 mC (SD=244.9) | 360.5 mC (SD=163.7) |
| Mean current | 1.7 A (SD=17.8) | 6.0 A (SD=47.7) |
| Mean stimulus duration | 7.8 s (SD=3.0) | 10.3 s (SD=32.8) |
| Mean frequency | 63.9 Hz (SD=15.9) | 52.2 Hz (SD=21.3) |
| Mean pulse width | 1.2 ms (SD=14.1) | 3.5 ms (SD=25.2) |
| Mean static impedance | 412.9 Ω (SD=173.6) | 398.8 Ω (SD=121.0) |
| Mean dynamic impedance | 149.0 Ω (SD=27.4) | 150.1 Ω (SD=33.3) |
| Mean motor seizure duration | 9.4 s (SD=9.8) | 13.7 s (SD=43.0) |
| Mean EEG seizure duration | 22.1 s (SD=19.7) | 20.9 s (SD=20.2) |
| **Third Stimulation** | | |
| Total procedures | 14 | 8 |
| Mean percentage energy | 59.6% (SD=34.3) | 66.4% (SD=37.6) |
| Mean charge | 301.8 mC (SD=173.2) | 336.0 mC (SD=190.4) |
| Mean current | 0.9 A (SD=0.0) | 0.9 A (SD=0.0) |
| Mean stimulus duration | 11.5 s (SD=18.4) | 6.7 s (SD=1.9) |
| Mean frequency | 45.0 Hz (SD=21.0) | 42.1 Hz (SD=26.1) |
| Mean pulse width | 0.5 ms (SD=0.0) | 0.5 ms (SD=0.0) |
| Mean static impedance | 382.9 Ω (SD=102.5) | 361.2 Ω (SD=95.8) |
| Mean dynamic impedance | 145.0 Ω (SD=17.0) | 137.5 Ω (SD=21.2) |
| Mean motor seizure duration | 14.5 s (SD=33.6) | 3.9 s (SD=5.1) |
| Mean EEG seizure duration | 25.6 s (SD=35.8) | 31.2 s (SD=56.5) |
| **Fourth Stimulation** | | |
| Total procedures | 2 | 2 |
| Mean percentage energy | 32.5% (SD=10.6) | 57.5% (SD=60.1) |
| Mean charge | 165.9 mC (SD=54.4) | 291.8 mC (SD=305.4) |
| Mean current | 0.9 A (SD=0.0) | 0.9 A (SD=0.0) |
| Mean stimulus duration | 7.2 s (SD=0.4) | 6.1 s (SD=2.7) |
| Mean frequency | 25.0 Hz (SD=7.1) | 45.0 Hz (SD=35.4) |
| Mean pulse width | 0.5 ms (SD=0.0) | 0.5 ms (SD=0.0) |
| Mean static impedance | 330.0 Ω (SD=28.3) | 390.0 Ω (SD=28.3) |
| Mean dynamic impedance | 140.0 Ω (SD=14.1) | 140.0 Ω (SD=0.0) |
| Mean motor seizure duration | 1.5 s (SD=2.1) | 13.5 s (SD=19.1) |
| Mean EEG seizure duration | 14.0 s (SD=1.4) | 49.0 s (SD=62.2) |

*^a^ Hyperventilation data were available for 1,630 of 1,685 autism procedures and 1,116 of 1,138 non-autism procedures; percentages are computed from evaluable cases. Stimulation parameter means and standard deviations are computed from procedures with non-missing values for each parameter.*
