# Supplementary material for: Electroconvulsive Therapy for Catatonia in Autistic and Non-Autistic Patients: An Observational Study on Course, Efficacy, Aggression, and Self-Injury Outcomes
Source: Autism Res. Author manuscript; Available in PMC 2026 Sep 27. (PMC13616504; doi:10.1002/aur.70362)
Supplement: Supplementary file 3 [file NIHMS2209361-supplement-Supplemental_Table_2.docx]

**Supplemental Table 2: Medications Administered During Electroconvulsive Therapy**

| **Anesthetic** | **Autism N (%)** | **Autism mean dose** | **Non-autism N (%)** | **Non-autism mean dose** |
| --- | --- | --- | --- | --- |
| Methohexital | 1,665 (98.8%) | 90.9 mg (SD=34.6) | 1,115 (98.0%) | 89.3 mg (SD=41.1) |
| Succinylcholine | 1,635 (97.0%) | 64.8 mg (SD=31.0) | 1,057 (92.9%) | 62.7 mg (SD=21.1) |
| Ondansetron | 933 (55.4%) | 4.1 mg (SD=4.0) | 223 (19.6%) | 3.9 mg (SD=0.4) |
| Ketorolac | 676 (40.1%) | 24.3 mg (SD=7.6) | 367 (32.2%) | 25.6 mg (SD=6.9) |
| Glycopyrrolate | 529 (31.4%) | 0.3 mg (SD=1.3) | 187 (16.4%) | 0.2 mg (SD=0.1) |
| Dexmedetomidine | 247 (14.7%) | — | 62 (5.4%) | — |
| Caffeine | 196 (11.6%) | 332.6 mg (SD=135.4) | 67 (5.9%) | 464.8 mg (SD=229.8) |
| Propofol | 90 (5.3%) | 53.8 mg (SD=33.4) | 72 (6.3%) | 83.1 mg (SD=72.2) |
| Rocuronium | 36 (2.1%) | 28.9 mg (SD=17.6) | 76 (6.7%) | 28.7 mg (SD=5.5) |
| Ketamine (IV anesthetic) | 3 (0.2%) | 66.7 mg (SD=28.9) | 1 (0.1%) | — |

*Values are the number and percentage of procedures in which each medication was administered and the mean dose among administrations with available dosing data. Dashes indicate that dosing data were unavailable. Propofol use includes all documented administrations and is not limited to treatment of prolonged seizures.*
