# Supplementary material for: Electroconvulsive Therapy for Catatonia in Autistic and Non-Autistic Patients: An Observational Study on Course, Efficacy, Aggression, and Self-Injury Outcomes
Source: Autism Res. Author manuscript; Available in PMC 2026 Sep 27. (PMC13616504; doi:10.1002/aur.70362)
Supplement: Supplementary file 4 [file NIHMS2209361-supplement-Supplemental_Table_3.docx]

**Supplemental Table 3: Comorbidities for Autistic Patients with Catatonia**

|  | **N** | **%** |
| --- | --- | --- |
| N | 43 |  |
| **Medical Co-morbidities** | | |
| Epilepsy | 8 | 18.6 |
| PANS/PANDAS | 4 | 9.3 |
| Lennox-Gastaut syndrome | 3 | 7.0 |
| Obstructive sleep apnea | 3 | 7.0 |
| Cerebral palsy | 3 | 7.0 |
| Hypothyroidism | 2 | 4.7 |
| COVID-19 | 2 | 4.7 |
| Delirium | 1 | 2.3 |
| Type 1 diabetes mellitus | 1 | 2.3 |
| Retinitis pigmentosa | 1 | 2.3 |
| Hirschsprung’s disease | 1 | 2.3 |
| Eosinophilic esophagitis | 1 | 2.3 |
| Chiari malformation | 1 | 2.3 |
| Congenital hydrocephalus | 1 | 2.3 |
| Seronegative autoimmune encephalitis | 1 | 2.3 |
| Sacral ganglioneuroblastoma | 1 | 2.3 |
| Neurodegeneration with brain iron accumulation | 1 | 2.3 |
| La Crosse encephalitis | 1 | 2.3 |
| **Psychiatric and Neurodevelopmental Co-morbidities** | | |
| Intellectual disability | 38 | 88.4 |
| Attention-deficit hyperactivity disorder | 11 | 25.6 |
| Obsessive-compulsive disorder | 8 | 18.6 |
| Bipolar disorder | 5 | 11.6 |
| Unspecified mood disorder | 4 | 9.3 |
| Generalized anxiety disorder | 3 | 7.0 |
| Pervasive developmental disorder | 3 | 7.0 |
| Post-traumatic stress disorder | 2 | 4.7 |
| Tourette’s syndrome / tic disorder | 2 | 4.7 |
| Substance-induced psychotic disorder | 1 | 2.3 |
| Schizoaffective disorder, bipolar type | 1 | 2.3 |
| Unspecified psychosis | 1 | 2.3 |
| Unspecified anxiety disorder | 1 | 2.3 |
| Unspecified schizoaffective disorder | 1 | 2.3 |
| Substance use disorder | 1 | 2.3 |
| **Genetic Co-morbidities** | | |
| SLC6A1, NEUROD2, CACNA2D2, KDM2B gene mutations | 1 | 2.3 |
| SLC6A1, CACNA2D2, GABBR2, RELN gene mutations | 1 | 2.3 |
| Down syndrome | 1 | 2.3 |
| SCN2A | 1 | 2.3 |
| SYNGAP1-related intellectual disability | 1 | 2.3 |
| SCAPER variant | 1 | 2.3 |
| CHD8-gene related disorder | 1 | 2.3 |
| Biotin-thiamine-responsive basal ganglia disease | 1 | 2.3 |
| Phelan-McDermid syndrome | 1 | 2.3 |
| Williams syndrome | 1 | 2.3 |
| ACSL4 and PCDH11X gene mutations | 1 | 2.3 |

Percentages were calculated using the full autism cohort denominator (N=43). Categories were not mutually exclusive.
