# Supplementary material for: Electroconvulsive Therapy for Catatonia in Autistic and Non-Autistic Patients: An Observational Study on Course, Efficacy, Aggression, and Self-Injury Outcomes
Source: Autism Res. Author manuscript; Available in PMC 2026 Sep 27. (PMC13616504; doi:10.1002/aur.70362)
Supplement: Supplementary file 5 [file NIHMS2209361-supplement-Supplemental_Table_4.docx]

**Supplemental Table 4: Comorbidities in Non-Autistic Patients with Catatonia**

|  | **N** | **%** |
| --- | --- | --- |
| N | 67 |  |
| **Medical Comorbidities** | | |
| Hypertension | 10 | 14.9 |
| Diabetes | 6 | 9.0 |
| Obstructive sleep apnea | 5 | 7.5 |
| Delirium | 5 | 7.5 |
| Hypothyroidism | 5 | 7.5 |
| Hyperlipidemia | 5 | 7.5 |
| Epilepsy | 5 | 7.5 |
| Cerebral palsy | 3 | 4.5 |
| Anti-NMDA receptor encephalitis | 2 | 3.0 |
| Seronegative autoimmune encephalitis | 2 | 3.0 |
| Chronic heart failure | 2 | 3.0 |
| Thromboembolic stroke | 2 | 3.0 |
| PANS/PANDAS | 1 | 1.5 |
| COVID-19 | 1 | 1.5 |
| Growth hormone deficiency | 1 | 1.5 |
| Endometriosis | 1 | 1.5 |
| Severe protein-calorie malnutrition | 1 | 1.5 |
| Microcephaly | 1 | 1.5 |
| Right middle cerebral artery stroke | 1 | 1.5 |
| Vascular dementia | 1 | 1.5 |
| Refeeding syndrome | 1 | 1.5 |
| **Psychiatric and Neurodevelopmental Comorbidities** | | |
| Major depressive disorder | 17 | 25.4 |
| Schizophrenia | 14 | 20.9 |
| Bipolar disorder | 13 | 19.4 |
| Substance use disorder | 9 | 13.4 |
| Intellectual disability | 8 | 11.9 |
| Schizoaffective disorder, bipolar type | 7 | 10.4 |
| Unspecified psychosis | 7 | 10.4 |
| Post-traumatic stress disorder | 6 | 9.0 |
| Generalized anxiety disorder | 4 | 6.0 |
| Attention-deficit hyperactivity disorder | 3 | 4.5 |
| Unspecified anxiety disorder | 3 | 4.5 |
| Unspecified trauma and related disorder | 2 | 3.0 |
| Postpartum psychosis / depression | 2 | 3.0 |
| Obsessive-compulsive disorder | 1 | 1.5 |
| Schizoaffective disorder, depressed type | 1 | 1.5 |
| Unspecified mood disorder | 1 | 1.5 |
| Schizophreniform disorder | 1 | 1.5 |
| Pervasive developmental disorder | 1 | 1.5 |
| **Genetic and Neurodegenerative Comorbidities** | | |
| Down syndrome | 2 | 3.0 |
| Huntington’s disease | 1 | 1.5 |
| Harboyan syndrome | 1 | 1.5 |
| Darier disease | 1 | 1.5 |
| Alport syndrome | 1 | 1.5 |

*Percentages were calculated using the full non-autism cohort denominator (N=67). Categories were not mutually exclusive.*
