# Supplementary material for: Electroconvulsive Therapy for Catatonia in Autistic and Non-Autistic Patients: An Observational Study on Course, Efficacy, Aggression, and Self-Injury Outcomes
Source: Autism Res. Author manuscript; Available in PMC 2026 Sep 27. (PMC13616504; doi:10.1002/aur.70362)
Supplement: Supplementary file 6 [file NIHMS2209361-supplement-Supplemental_Table_5.docx]

**Supplemental Table 5: Pharmacotherapy at the Time of Electroconvulsive Therapy Consultation**

|  | **Autism and catatonia (N = 43)** | | | **Non-autism and catatonia (N = 67)** | | |
| --- | --- | --- | --- | --- | --- | --- |
|  | N | % | Mean daily dose | N | % | Mean daily dose |
| **Antipsychotics** | | | | | | |
| Risperidone | 7 | 16.3 | 1.9 mg | 8 | 11.9 | 2.8 mg |
| Aripiprazole | 7 | 16.3 | 9.8 mg | 5 | 7.5 | 5.7 mg |
| Olanzapine | 4 | 9.3 | 15.0 mg | 23 | 34.3 | 14.5 mg |
| Quetiapine | 3 | 7.0 | 241.7 mg | 3 | 4.5 | 200.0 mg |
| Chlorpromazine | 3 | 7.0 | 62.5 mg | 1 | 1.5 | 200.0 mg |
| Clozapine | 2 | 4.7 | 125.0 mg | 2 | 3.0 | 156.2 mg |
| Paliperidone | 2 | 4.7 | 4.5 mg | 0 | 0 | — |
| Ziprasidone | 2 | 4.7 | — | 0 | 0 | — |
| Haloperidol | 1 | 2.3 | — | 6 | 9.0 | 17.2 mg |
| Lurasidone | 0 | 0 | — | 1 | 1.5 | 80.0 mg |
| **Mood stabilizers and antiseizure medications** | | | | | | |
| Lamotrigine | 8 | 18.6 | 228.6 mg | 3 | 4.5 | 100.0 mg |
| Lithium | 7 | 16.3 | 1,071.4 mg | 6 | 9.0 | 650.0 mg |
| Valproic acid | 6 | 14.0 | 1,083.3 mg | 7 | 10.4 | 673.3 mg |
| Oxcarbazepine | 5 | 11.6 | 990.0 mg | 0 | 0 | — |
| Gabapentin | 4 | 9.3 | — | 1 | 1.5 | — |
| Topiramate | 2 | 4.7 | 262.5 mg | 1 | 1.5 | 100.0 mg |
| Zonisamide | 1 | 2.3 | — | 1 | 1.5 | — |
| Levetiracetam | 0 | 0 | — | 1 | 1.5 | 3,000.0 mg |
| **Benzodiazepines** | | | | | | |
| Clonazepam | 19 | 44.2 | — | 4 | 6.0 | — |
| Lorazepam | 18 | 41.9 | — | 53 | 79.1 | — |
| Diazepam | 2 | 4.7 | — | 2 | 3.0 | — |
| Clobazam | 1 | 2.3 | — | 0 | 0 | — |
| Lorazepam + clonazepam | 1 | 2.3 | — | 2 | 3.0 | — |
| **Antidepressants** | | | | | | |
| Fluoxetine | 5 | 11.6 | 40.0 mg | 1 | 1.5 | 10.0 mg |
| Trazodone | 5 | 11.6 | 125.0 mg | 10 | 14.9 | 65.0 mg |
| Sertraline | 4 | 9.3 | 72.5 mg | 8 | 11.9 | 96.9 mg |
| Escitalopram | 3 | 7.0 | 16.7 mg | 3 | 4.5 | 10.0 mg |
| Fluvoxamine | 3 | 7.0 | 150.0 mg | 0 | 0 | — |
| Duloxetine | 1 | 2.3 | 60.0 mg | 2 | 3.0 | 90.0 mg |
| Citalopram | 1 | 2.3 | 20.0 mg | 0 | 0 | — |
| Mirtazapine | 0 | 0 | — | 5 | 7.5 | 10.5 mg |
| Venlafaxine | 0 | 0 | — | 1 | 1.5 | 225.0 mg |
| **ADHD- and cognition-related medications** | | | | | | |
| Guanfacine | 12 | 27.9 | 2.8 mg | 1 | 1.5 | 6.0 mg |
| Memantine | 10 | 23.3 | 16.5 mg | 2 | 3.0 | 12.5 mg |
| Methylphenidate | 3 | 7.0 | 25.0 mg | 0 | 0 | — |
| Clonidine | 2 | 4.7 | 0.2 mg | 1 | 1.5 | — |
| Atomoxetine | 1 | 2.3 | 80.0 mg | 0 | 0 | — |

*Percentages were calculated using the full cohort denominator. Patients could receive medications from more than one category. Mean daily doses were calculated among patients with available dosing data; when only one dose was available, the displayed value represents that single observation. Dashes indicate unavailable dosing data or no medication use. Medication rows were not mutually exclusive. The combination benzodiazepine row identifies concurrent lorazepam and clonazepam use.*
