# Supplementary material for: Electroconvulsive Therapy for Catatonia in Autistic and Non-Autistic Patients: An Observational Study on Course, Efficacy, Aggression, and Self-Injury Outcomes
Source: Autism Res. Author manuscript; Available in PMC 2026 Sep 27. (PMC13616504; doi:10.1002/aur.70362)
Supplement: Supplementary file 7 [file NIHMS2209361-supplement-Supplemental_Table_6.docx]

**Supplemental Table 6: Electroconvulsive Therapy Technique Parameters by Calendar Year**

| **Parameter** | **2022** | **2023** | **2024** | **2025** | **2026** | **p for trend^a^** |
| --- | --- | --- | --- | --- | --- | --- |
| Procedures, n | 33 | 203 | 894 | 1,227 | 466 |  |
| **Binary parameters (% of procedures)** | | | | | | |
| ≥2 stimulations^b^ | 3.0 | 12.8 | 17.6 | 22.0 | 29.2 | <0.001 |
| IM ketamine for IV access | 0.0 | 28.6 | 20.7 | 12.1 | 21.0 | 0.020 |
| **Continuous parameters (mean, first stimulation)^c^** | | | | | | |
| Percentage energy (%) | 83.8 | 53.2 | 63.7 | 72.1 | 80.4 | <0.001 |
| Charge (mC) | 500.8 | 267.4 | 313.8 | 354.3 | 400.9 | <0.001 |
| EEG seizure duration (s) | 43.8 | 72.7 | 50.4 | 45.7 | 41.5 | <0.001 |
| Pulse width (ms) | 0.8 | 0.5 | 1.1 | 0.6 | 0.5 | 0.438 |

**^a^ P-values were obtained from logistic regression for binary outcomes and linear regression for continuous outcomes, with calendar year of procedure modeled as a numeric predictor.**

**^b^ The proportion of procedures with at least two stimulations increased monotonically across calendar years; the estimated odds ratio was 1.42 per year.**

**^c^ Annual means for percentage energy, charge, and EEG seizure duration were not uniformly monotonic. The 2022 estimates were based on 33 procedures.**
